# Quantification of amino acids and peptides in an ionic liquid based aqueous two-phase system by LC–MS analysis

**DOI:** 10.1186/s13568-018-0596-1

**Published:** 2018-04-25

**Authors:** Sebastian Oppermann, Christina Oppermann, Miriam Böhm, Toni Kühl, Diana Imhof, Udo Kragl

**Affiliations:** 1 HTT Biochemistry Diagnostic, Centogene AG, Am Strande 7, 18055 Rostock, Germany; 2 Institute of Chemistry, University of Rostock, Albert-Einstein-Str. 3A, 18059 Rostock, Germany; 3 Sanofi-Aventis Deutschland GmbH, Industriepark Höchst, 65926 Frankfurt am Main, Germany; 4 Pharmaceutical Chemistry I, Institute of Pharmacy, Rheinische Friedrich-Wilhelms-University, Brühler Strasse 7, 53119 Bonn, Germany

**Keywords:** LC–MS, Amino acids, Peptides, Ionic liquids, Aqueous two-phase system, Partitioning behavior

## Abstract

Aqueous two-phase systems (ATPS) occur by the mixture of two polymers or a polymer and an inorganic salt in water. It was shown that not only polymers but also ionic liquids in combination with inorganic cosmotrophic salts are able to build ATPS. Suitable for the formation of ionic liquid-based ATPS systems are hydrophilic water miscible ionic liquids. To understand the driving force for amino acid and peptide distribution in IL-ATPS at different pH values, the ionic liquid Ammoeng 110™ and K_2_HPO_4_ have been chosen as a test system. To quantify the concentration of amino acids and peptides in the different phases, liquid chromatography and mass spectrometry (LC–MS) technologies were used. Therefore the peptides and amino acids have been processed with EZ:faast™-Kit from Phenomenex for an easy and reliable quantification method even in complex sample matrices. Partitioning is a surface-dependent phenomenon, investigations were focused on surface-related amino acid respectively peptide properties such as charge and hydrophobicity. Only a very low dependence between the amino acids or peptides hydrophobicity and the partition coefficient was found. Nevertheless, the presented results show that electrostatic respectively ionic interactions between the ionic liquid and the amino acids or peptides have a strong impact on their partitioning behavior.

## Introduction

During the past few decades ionic liquids have become an essential part in chemical and biochemical research (Wasserscheid and Welton 2008). Ionic liquids are organic salts with unique physical properties, for example high thermal stability, low viscosity and a negligible vapor pressure. Below 100 °C, ionic liquids exist in a liquid state (Seddon 1997). These properties depend on their structure. Steric effects and charge delocalization of their ions hinder the formation of a stable crystal lattice (Kyte and Dolittle 1982). Already low thermal energy is sufficient to overcome the lattice energy and to break up the solid crystalline structure (Kyte and Dolittle 1982). Ethyl ammonium nitrate was the first known ionic liquid and was described by Walden in 1914 (Walden 1914). A number of various ionic liquids have been described in the last decades. Some examples of water miscible ionic liquids are shown in Table 1 (Wasserscheid and Welton 2008).

**Table 1:**
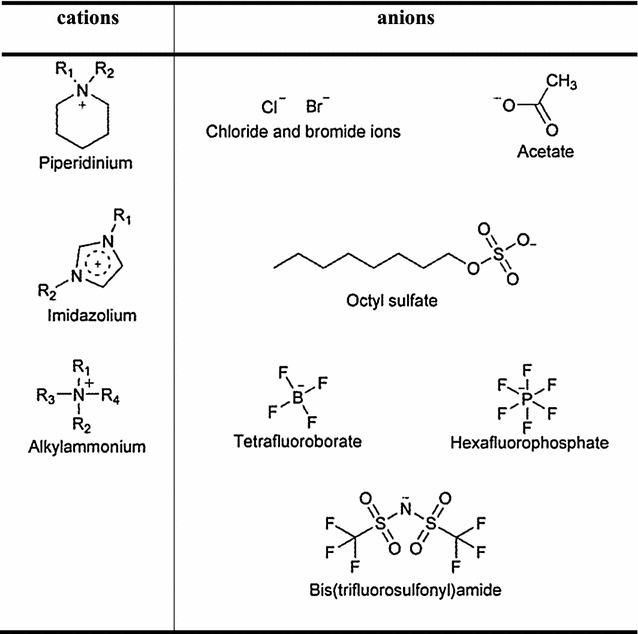
Examples for ionic liquids

They have anions such as [BF_4_], [PF_6_], [Tf_2_N] but also halides like chloride and bromide ions (Wasserscheid and Welton 2008). The most common cations were based on 1-ethyl-3-methylimidazolium, but also piperidinium based as well as quaternary ammonium cations are described (Wilkes and Zaworotko 1992). These ionic liquids can also be applied in aqueous two-phase systems (ATPS) (Dreyer and Kragl 2008; Dreyer et al. 2009; Kragl et al. 2002). Historically ATPS occur by the mixture of two polymers, and also of a polymer and an inorganic salt in water (Flory 1953). Phase separation in ATPS occurs when the enthalpy of the interaction of the polymer molecules is higher than the loss of entropy due to the phase separation. It was shown that not only polymers but also ionic liquids in combination with inorganic cosmotrophic salts are able to build ATPS (Bonhote et al. 1996; He et al. 2005; Holbrey and Seddon 1999; Li et al. 2005). Suitable for the formation of ionic liquid-based ATPS are water miscible ionic liquids (Kohno et al. 2011; 2012). In 2003 Rogers and coworkers investigated the ability of cosmotrophic salts (i.e. K_3_PO_4_) to salt-out hydrophilic ionic liquids such as [C_4_mim][Cl] and [N_4444_][Cl] (Gutowski et al. 2003). They were the first scientists who investigated the use of ionic liquids for the generation of ATPS and reported the phase diagrams of ionic liquid-based ATPS (Gutowski et al. 2003). The application of ionic liquid-based ATPS for the extraction of testosterone and epitestosterone in human urine or the extraction of major opium alkaloids in *Pericarpium papaveris* using [C_4_mim][Cl]/K_2_HPO_4_ systems was first achieved by Liu and coworkers (He et al. 2005; Li et al. 2005). Du et al. were the first to report the extraction of proteins by ionic liquid-based ATPS (Du et al. 2007).

**Fig. 1:**
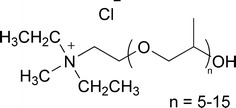
Structure of Ammoeng 110™

Another positive effect of ATPS is the short time of phase separation. For ionic liquid-based ATPS phase separation is completed within a couple of seconds up to a few minutes (Dreyer and Kragl 2008). For this study the ionic liquid Ammoeng 110™ (Fig. 1) and K_2_HPO_4_ were chosen as a test system to understand the driving force for amino acid and peptide distribution at different pH values.

To quantify the amount of amino acids and peptides in the different phases, LC–MS technologies have been used. Therefore the peptides and amino acids were processed with the EZ:faast™ technology from Phenomenex to provide an easy, reliable and reproducible quantification method (Dziagwa-Becker et al. 2015; Phenomenex 2005). The Phenomenex EZ:faast™ amino acid analysis kit is based on solid-phase extraction (SPE), thus saving time on prior removal of disturbing solvents such as ionic liquids. This kit allows a reliable and reproducible determination of amino acids and proteins even in complex sample matrices. Due to their special behavior ionic liquids, which resemble high salt concentrations rather than organic solvents, they are a challenge to the analytical procedure, which can be solved with the EZ:faast™ kit.

## Materials and methods

### Chemicals

Amino acids were purchased from Sigma Aldrich as kit containing 22 standards. Peptides were produced by a standard solid-phase protocol using the Fmoc-strategy and HBTU as coupling reagent and purified by semi-preparative RP-HPLC (> 95% HPLC-pure) prior to use. The internal standard solution, eluting medium component I and II, organic solution I and II and the standard solution mixtures SD 1 and SD 2 are components of the EZ:faast™ amino acid analysis kit from Phenomenex (Aschaffenburg, Germany). The EZ:faast™ amino acid analysis kit from Phenomenex includes all materials which are needed for the sample preparation, calibration and derivatization. The LC–MS Chromasolv^®^ grade solvents, methanol with 0.1% formic acid and water with 0.1% formic acid were obtained by Fluka (since 2005 Sigma-Aldrich, Switzerland). The ionic liquid Ammoeng 110™ was provided by Solvent Innovation GmbH (Köln, Germany) which is now part of Merck KGaA (Darmstadt, Germany). K_2_HPO_4_, KH_2_PO_4_ and HCl solution were purchased at Merck KGaA (Darmstadt, Germany).

### IL-ATPS

To determine the distribution ratio (D) in an ATPS a two-phase system was built. In the beginning, two buffer solutions have been prepared: (i) buffer I with a pH value of 6.0, and (ii) buffer II with a pH value of 7.0. For each buffer 44 g K_2_HPO_4_ and 36 g KH_2_PO_4_ have been weighed into a 500 mL measuring flask. Buffer I was filled up with ultrapure water to a total weight of 300 g. Next the buffer was adjusted to pH 6.0 with 20% HCl solution and filled up with ultrapure water to a total weight of 400 g. Buffer II with pH 7.0 was directly filled up with ultrapure water to a total weight of 400 g. The method ensured that no different ionic species were present in the mixtures. Chloride as anion is introduced by the ionic liquid itself. The small difference in ionic strength is neglected.

Afterwards 10–15 mg of each amino acid and peptide to be distributed at both pH values have been measured in 15 mL vials. For each amino acid respectively peptide, one of the vials was filled up with buffer I and the other with buffer II so that each vial had a total volume of 11 mL. Next the samples have been mixed by vortexing to dissolve the substances until the solution was clear. The amount of the amino acids and peptides had been selected to have a maximum of 20 nmol in each vial. This amount of substance was optimized to be detected by LC–MS measurement.

All the measurements were done in duplicates. For each sample two 4 mL vials have been used to measure 0.60–0.65 g of ionic liquid Ammoeng 110™ into each vial. Afterwards every vial was filled up with one of the amino acid/peptide solutions to a final mass of 4 g. All samples have been mixed by vortexing for 45 s. After 5 min the phase separation was finished.

### Volume determination

In order to determine distribution ratios (D) it is crucial to know the volume of each phase. The volumes (V) of the upper and lower phases of the IL-ATPS were calculated using the following mathematical formulae (Kemnitz 2010):

1 $$V = \frac{\pi }{4}*d^{2} *(h - b)$$

2 $$V_{upper} = V_{total} - V_{lower}$$

In order to avoid random errors, a digital caliper from Mitutoyo was used to measure and average the height (h) of the liquid level of the lower and total phases (in triplicates). The diameter of the vial (d) was 1.2785 cm and the ground level (b) was 0.09475 cm.

### Internal and calibration standards

For quantification purposes the standard solutions SD 1 and SD 2, which were included in the EZ:faast™ amino acid analysis kit from Phenomenex, were used. Standards 1 and 2 are mixtures of most amino acids occurring in plasma and were used throughout this work. Standard 1 contains 27 amino acids in 0.05 N HCl solution with a concentration of 200 µmol/L. Standard 2 contains only the three amino acids asparagine, glutamine and tryptophan in the same concentration. These are not stable in acidic solution. Calculation and calibration are based on an internal standard method with three different calibration levels. Calibration level 1 consists of 10 µL SD 1 and SD 2 plus 100 µL internal standard solution. Calibration level 2 consists of 50 µL SD 1 and SD 2 plus 100 µL internal standard solution and calibration level 3 contained 100 µL SD 1, SD 2 and internal standard. The kit contains as internal standard an amino acid mixture of homoarginine (HARG), methionine-d3 (Met-d3) and homophenylalanine (HPHE). The concentration in calibrators and samples was set to 200 µmol/L. As recommended in the EZ:faast™ User’s Manual of Phenomenex the HARG was used as internal standard for early eluting amino acids like arginine (Arg), Met-d3 for the middle eluting amino acids from serine (Ser) to tryptophan (Trp) and HPHE for the late eluting amino acids like leucine (Leu) and tyrosine (Tyr) (Phenomenex 2005).

### Preparing the eluting medium for solid phase extraction (SPE)

The eluent for the standards and samples was a combination of three parts of the eluting medium component I (sodium hydroxide) and two parts of the eluting medium component II (n-propanol) which were mixed quickly in a capped vial of appropriate size. The eluent was prepared freshly each day, and the required volume depended on the number of samples (200 µL/sample).

### Preparing the HPLC mobile phase

The HPLC mobile phase was a mixture of water with 0.1% formic acid and methanol with 0.1% formic acid 1:2, v/v.

### Peptide hydrolysis

The hydrolysis reagents and supplies were not included in the EZ:faast™ Kit. An acidic hydrolysis with 6 M HCl in liquid phase was used.

100 µL of each, upper and lower phase, of the peptide-containing IL-based ATPS was transferred into 1.5 mL Eppendorf tubes. Afterwards every sample was mixed with 900 µL of 6 M HCl solution and incubated for 20 h at 110 °C. For the EZ:faast™ procedure it is necessary to have a pH value between 1.5 and 5.0 for the mixture. To achieve this 100 µL of each sample was mixed with 150 µL saturated Na_2_CO_3_ solution.

### Sample preparation by EZ:faast™ solid phase extraction

100 μL of each sample and 100 μL of the internal standard solution were pipetted into a vial. Next, a sorbent tip was attached to a 1.5 mL syringe. The tip was immersed and the solution in the sample preparation vial passed through the sorbent tip by slowly pulling back the syringe piston in small steps (∼ 1 min). Afterward 200 μL of n-propanol (washing solution included in the EZ:faast™ Kit) were transferred into the same vial and then passed slowly through the sorbent tip into the syringe barrel. The liquid was drained from the sorbent bed by pulling air through the sorbent tip. At that point, the latter was detached and left in the vial. Afterwards 200 μL of the freshly prepared elution medium was filled in the same vial. After pulling back the piston of the 0.6 mL syringe halfway up, the syringe was attached to the sorbent tip which was used before. Then, the sorbent was flushed out with the eluting medium. After this the liquid was ejected so that all sorbent particles in the tip were expelled into the sample preparation vial.

### Derivatization by EZ:faast™

The derivatization was performed in the same vial. For this purpose, the Drummond Dialamatic micro dispenser was used to transfer 50 μL of the included reagent 4 (mixture of chloroform and the derivatization substance) into the vial. Next, the solutions were mixed by repeated vortexing for 5–6 s. The reaction was allowed to proceed for at least 1 min. The emulsion gradually separated into two layers. Then the liquid was mixed again by vortexing the vial for 5 s and the reaction was allowed to proceed for another minute. Afterward 100 μL of iso-octane (included in the EZ:faast™ Kit) were transferred with the micro dispenser and mixed for 5 s. Then, the reaction was left to proceed for one more minute, and the emulsion separated into two phases. The upper (organic) phase contained the derivatized amino acids to be analyzed by LC–MS. The upper phase was transferred into another vial and evaporated to dryness in a gentle steam of argon at room temperature in less than 10 min. Afterwards the analyte has been dissolved in 100 µL of the HPLC mobile phase to be able to perform LC–MS analysis.

### LC–MS

The standards and samples were analyzed on a Finnigan Surveyor high performance liquid chromatography (HPLC) system equipped with a Finnigan linear trap quadrupole (LTQ) mass spectrometer (Thermo Scientific, Germany). The chromatographic separation was performed on a EZ:faast™ AAA-MS HPLC column which is part of EZ:faast™ amino acid analysis kit by Phenomenex. The temperature of the column was set to 35 °C. The mobile phase consisted of a gradient eluting system. Solvent A was methanol with 0.1% formic acid (LC–MS Chromasolv^®^, Fluka) and solvent B was water with 0.1% formic acid (LC–MS Chromasolv^®^, Fluka). Elution of the standards and samples was achieved with the following solvent gradient: 68% A to 83% A (22 min), 83% A to 68% A (0.01 min) and 68% A isocratic (27.99 min). The flow rate of the mobile phase was set to 0.15 mL/min, and the volume of injection was 2 µL.

The amino acids were identified by ion trap technology and the mass spectrometric ionization was performed with electrospray (Heimer et al. 2014). MS spectra were recorded consecutively in one segment with one full scan event in the range of m/z 100.00–650.00. The scan event was conducted in a positive ion mode with a skimmer induced dissociation of 35.00. All data was evaluated and interpreted with Xcalibur Software (Thermo Scientific, Dreieich, Germany).

### Amino acid quantification and calculation of the distribution ratio

First the ratio between the area below the peak of the internal standard and the area below the peak of the analyte in the chromatogram was calculated. The concentration of the analyte was calculated using the slope of a calibration curve and the determined ratio.

Afterwards, the distribution ratio (D) was calculated as the ratio between the concentration (c) of the amino acid in the upper phase to the concentration of the amino acid in the lower phase (Leo et al. 1971):

3 $$D = \frac{{C_{upper\;phase} }}{{C_{lower\;phase} }}$$

Then, the decadic logarithm of the distribution ratio was calculated (logD-value) Therefore, analytes accumulating in the ionic liquid-rich upper phase have a positive logD-value, analytes accumulating in the lower phase show a negative logD-value.

## Results

### Amino acid distribution in ionic liquid-ATPS at different pH values

**Fig. 2:**
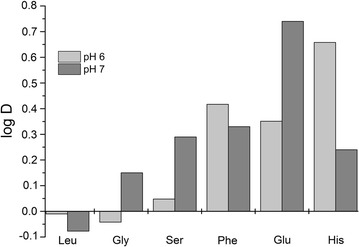
Distribution ratios of free amino acids in IL-ATPS at pH 6.0 and 7.0

The partitioning behavior of six different amino acids was investigated. Structures and pI values of the investigated amino acids are given in Table 2 (Hardy 1985; Jakubke and Jeschkeit 1982; Kyte and Dolittle 1982). The systems pH values were set to 6.0 respectively 7.0 to investigate the partitioning behavior of glycine, leucine and phenylalanine near their isoelectric point (pH 6.0) and in neutral environment (pH 7.0). The logarithms of the distribution ratios of the tested free amino acids are shown in Fig. 2.

**Table 2:** Structure and pI values of the investigated amino acids

Amino acid	Structure	One-letter code	Three-letter code	pI	Hydrophobicity of the side chain
Glutamic acid		E	Glu	3.24^a^	− 3.5^c^
Glycine		G	Gly	5.97^a^	− 0.4^c^
Histidine		H	His	7.59^a^	− 3.2^c^
Leucine		L	Leu	5.98^a^	3.8^c^
Phenylalanine		F	Phe	5.48^b^	2.8^c^
Serine		S	Ser	5.68^a^	− 0.8^c^

^a^ Taken from Hardy (1985)

^b^ Taken from Jakubke and Jeschkeit (1982)

^c^ Taken from Kyte and Dolittle (1982)

It is obvious that phenylalanine (Phe), glutamic acid (Glu) and histidine (His) are strongly enriched in the ionic liquids-containing upper phase of the ATPS at both pH values. The distribution ratio of glutamic acid at pH 7.0 is much higher than at pH 6.0. Histidine on the other hand has a higher distribution ratio at the lower pH value of the system. The impact of the pH value of the system on the distribution of phenylalanine is not as strong as on His or Glu. Nevertheless, it is enriched in the ionic liquid-containing upper phase of the ATPS. The other tested amino acids, i.e. leucine, serine and glycine, are distributed equally between the ionic liquid-rich upper and the phosphate-rich lower phase of the ATPS at pH 6.0. At a pH value of 7.0, glycine and serine get distributed more into the ionic liquid-rich upper phase of the ATPS. The effect of the pH value on the distribution of serine in the ATPS is even stronger than the effect on glycine. For leucine the distribution behavior was not measurable.

### Distribution of pentapeptides in ionic liquid-ATPS at different pH values

To further investigate the driving force for the distribution of amino acids and peptides in our ionic liquid-ATPS, six different pentapeptides of the general structure AAXAA (X = D, F, G, H, L, and S) were tested (Table 3). The isoelectric point (pI) of the different pentapeptides were calculated via the BACHEM Peptide Calculator (BACHEM 2016).

**Table 3:**
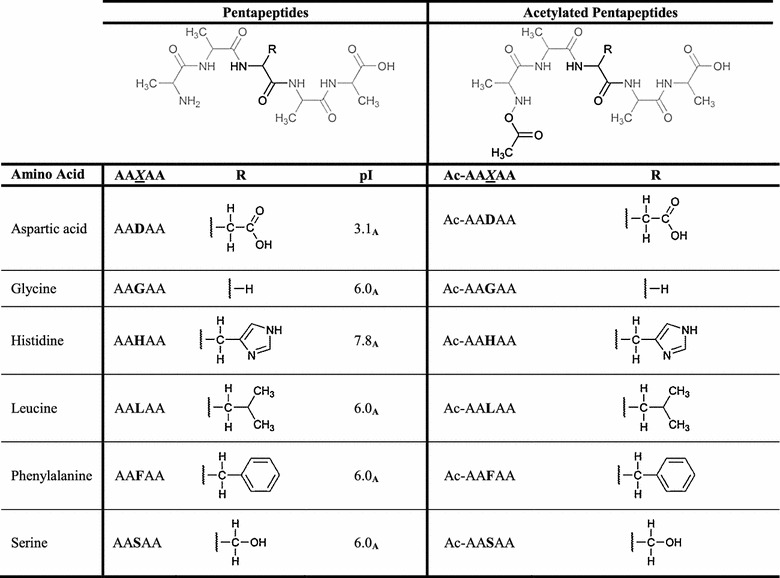
Structures and pI values of the investigated pentapeptides and structures of the investigated acetylated pentapeptides

R = side chain of the central amino acid

^a^ Calculated via peptide calculator from BACHEM(2016)

**Fig. 3:**
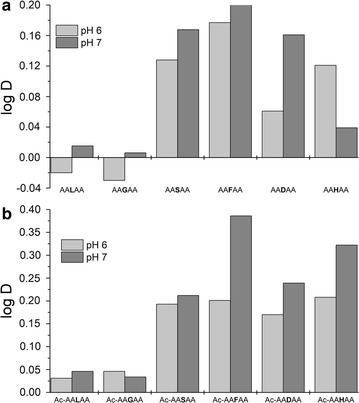
Distribution ratios of **a** pentapeptides and **b** acetylated pentapeptides at pH 6.0 and 7.0 (in IL-ATPS, *A* alanine, *L* leucine, *G* glycine; *S* serine; *F* phenylalanine; *D* aspartic acid; *H* histidine, *Ac* acetylated)

The central amino acid (X) differed in order to be able to monitor the impact of the individual side chain within a peptide backbone on the partitioning behavior (Table 3). To be able to compare the results of the pentapeptide distributions with the results of the experiments with the free amino acids, the same pH values of the ATPS were chosen (pH 6.0, pH 7.0). The logarithms of the distribution ratios of the tested pentapeptides were plotted (Fig. 3a).

It is obvious that phenylalanine—(AA**F**AA), serine—(AA**S**AA) and aspartic acid—(AA**D**AA) containing pentapeptides were concentrated in the ionic liquid-containing upper phase of the ATPS at both pH values. Also the His-containing pentapeptide (AA**H**AA) is enriched in the upper phase of the ATPS at pH 6.0 (Fig. 3a). At pH 7.0, accumulation of AA**H**AA in the upper phase was also detected, but not as strong as at pH 6.0 (Fig. 3a). The glycine- and the leucine-containing pentapeptides (AA**G**AA, AA**L**AA) were almost equally distributed between the two phases of the system at pH 6.0 and 7.0 (Fig. 3a). At pH 7.0, AA**G**AA and AA**L**AA were slightly more concentrated in the ionic liquid rich upper phase (Fig. 3a).

To overcome the influence of the charge of the alanine backbone of the pentapeptides on the partitioning behavior in IL-APS, N-terminally acetylated pentapeptides of the same general structure as described above were investigated (Table 3). The logarithms of the distribution ratios of the investigated acetylated pentapeptides were as well plotted (Fig. 3b). The phenylalanine- (Ac-AA**F**AA), serine- (Ac-AA**S**AA) and aspartic acid- (Ac-AA**D**AA) containing pentapeptides showed the same behavior at pH 6.0 and pH 7.0 like the corresponding not acetylated pentapeptides (Fig. 3). The Ac-AA**F**AA, the Ac-AA**D**AA as well as the Ac-AA**S**AA led to an accumulation of the analytes in the Ammoeng 110™-rich upper phase of the ATPS (Fig. 3b). Also, the His-containing pentapeptide (Ac-AA**H**AA) was enriched in the upper phase of the ATPS at pH 6.0 and pH 7.0 (Fig. 3b).

As observed before, the glycine- and the leucine-containing pentapeptides (Ac-AA**G**AA, Ac-AA**L**AA) were almost equally distributed between the two phases of the system at pH 6.0 and 7.0 (Fig. 3b). At both pH values, Ac-AA**G**AA and Ac-AA**L**AA were slightly more distributed in the ionic liquid rich upper phase (Fig. 3b).

## Discussion

The investigated amino acids have different structural requirements and influences to the partitioning behavior: leucine as an amino acid with a long aliphatic chain, glycine as the smallest natural amino acid, serine which has an alcohol group at its side chain, phenylalanine with a phenyl group, glutamic acid with a second carboxyl group and finally histidine which has an imidazole group (Table 2). The results show that there has to be a interaction between the cation of Ammoeng 110™ (AE110, IL) and the negatively charged carboxyl groups of the amino acids (Fig. 2). Because of this phenylalanine (Phe), glutamic acid (Glu) and histidine (His) are enriched in the ionic liquid containing upper phase of the ATPS at both pH values.

**Fig. 4:**
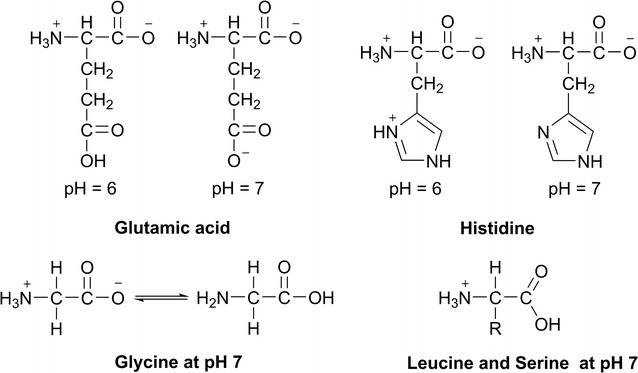
Charge state of different amino acids. R = side chain of the respective amino acid

The high distribution ratio of Glu at pH 7.0 could be due to the fact that the molecule is slightly more negatively charged at pH 7.0 compared to the pH 6.0. That could lead to a stronger ionic interaction between the cation of the ionic liquid and the amino acid at pH 7.0 compared to the charge of Glu at pH 6.0 (Fig. 4). At His there is a conversely effect. At pH 6.0 the His is protonated at its imidazole ring and therefore has an overall positive charge (Fig. 4) which leads to a higher distribution ratio at the lower pH value of the system (Fig. 2).

It can be assumed that the overall charge of the target molecule is crucial for the extraction into the AE110-rich upper phase of the ATPS due to ionic interactions between the ionic liquid molecules and the amino acid.

For the Phe the effect is not as strong as on His or Glu. But, it is also enriched in the ionic liquid containing upper phase of the ATPS. We suggest the electrostatic interactions between the electron-rich phenyl group of the amino acid and the cation of the ionic liquid Ammoeng 110™ to be the main driving force for that behavior. Also, a salting out effect due to the incompatibility of the phenyl group and the PO_4_^3−^-ions in the buffer-containing lower phase of the ATPS could enhance the concentration of phenylalanine in the upper phase.

The other tested amino acids, i.e. leucine, serine and glycine, occur in the betaine structure and have no net charge at a pH value 6.0 (Fig. 4).

Additionally, leucine has a hydrophobic side chain that hinders the ionic interaction between Ammoeng 110™ and the amino acid. At a pH value of 7.0, glycine and serine get distributed more into the IL-rich upper phase of the ATPS, while leucine was marginally higher concentrated in the phosphate buffer-rich lower phase (Fig. 2). At the higher pH value, the amino acids change their charge state, i.e. the relative charge of the molecule increases (Fig. 4).

The alcohol group of the side chain of serine is able to form hydrogen bonds with the cation of Ammoeng 110™. Because of this serine was enriched in the upper phase of the system and their distribution in the ATPS is even stronger than the effect of glycine. For leucine having a long aliphatic side chain (Table 2) the effect of the charge on the amino group on the distribution behavior was not measurable (Fig. 2).

For the driving force for the distribution of amino acids and peptides in our IL-ATPS, six different pentapeptides were tested (Fig. 3). The central amino acids were chosen in a way that the main groups of chemical structures occurring for amino acids are covered: (I) glycine as the simplest and most flexible amino acid without any side chain, (II) aliphatic, hydrophobic side chains (represented by leucine), (III) polar amino acids (represented by serine), (IV) aromatic amino acids (phenylalanine), (V) acidic residues (represented by aspartic acid), and finally (VI) basic amino acids (represented by histidine) (Table 3).

There has to be a strong interaction between the electron-rich phenyl group of the AA**F**AA-pentapeptide, the negatively charged carboxylic group of AA**D**AA as well as the hydroxygroup of the AA**S**AA-peptide and the cation of Ammoeng 110™ so that they were concentrated in the IL-containing upper phase of the ATPS at both pH values (Fig. 3a). At pH 7.0, accumulation of AA**H**AA in the upper phase was also detected, but not as strong as at pH 6.0 (Fig. 3a). Again the protonation of the imidazole group on the histidine side chain seems to have a big impact on the partitioning behavior of AAHAA in the IL-ATPS. As already described above, at pH 6.0 the histidine has one charge more compared to pH 7.0 (Fig. 4).

The overall charge of the target molecule is important for the extraction into the AE110-rich upper phase of the ATPS due to ionic interactions between the IL molecules and the pentapeptides.

The pentapeptides AA**G**AA and AA**L**AA were almost equally distributed between the two phases of the system at pH 6.0 and 7.0 (Fig. 3a). At pH 7.0, AA**G**AA and AA**L**AA were slightly more concentrated in the IL-rich upper phase (Fig. 3a). With glycine being the smallest amino acid with only hydrogen as a residue and leucine having an aliphatic side chain, the influence of the charge of the alanine backbone of the pentapeptides is much stronger compared to the other investigated pentapeptides. At pH 6.0, alanine, like glycine, is in a neutral state (Fig. 4). Therefore, it cannot interact as strong with the IL as at pH 7.0 when at least the N-terminus of the pentapeptide is highly protonated. Thus, the overall charge of the molecule has a major impact on the partitioning behavior.

To overcome the influence of the charge of the alanine backbone of the pentapeptides on the partitioning behavior in IL-ATPS, N-terminally acetylated pentapeptides of the same general structure as described above were investigated (Table 3). The logarithms of the distribution ratios of the investigated acetylated pentapeptides were as well plotted (Fig. 3b). The acetylated pentapeptides showed the same behavior at pH 6.0 and pH 7.0 like the corresponding not acetylated pentapeptides (Fig. 3). A strong interaction between the electron-rich phenyl group of the Ac-AA**F**AA, the negative charge of the carboxyl group of the Ac-AA**D**AA as well as the polar hydroxy group of Ac-AA**S**AA and the cation of the IL led to an accumulation of the analytes in the AE110-rich upper phase of the ATPS (Fig. 3b).

For both central amino acids **G** respectively **L**, being not charged or possessing an aliphatic side chain, it is very likely that the influence of hydrophobic interactions between Ammoeng 110™ and the peptide are not as important for the partitioning behavior than the electrostatic interactions, i.e. the overall charge of the molecule and therefore electrostatic/ionic interactions were obviously the main driving force for pentapeptide extraction into the Ammoeng 110™-rich upper phase of the ATPS.

To sum up our results, we observed that amino acids respectively pentapeptides that have side chains providing charged or chargeable functional groups like an imidazole ring (His) or hydroxy groups (Ser) get extracted into the IL-containing upper phase of the system. On the other hand, amino acids or pentapeptides with aliphatic or very small side chains like leucine and glycine were distributed almost equally between the two phases, when not getting charged like the free glycine at a pH value of 7.0. Particularly leucine and the leucine-containing pentapeptides showed insignificant changes in their partitioning behavior when the pH value altered. We obtained contrary results for the acetic amino acids aspartic acid and glutamic acid. It was possible to monitor a strong impact of the pH value of the system on the distribution ratio of the investigated amino acids respectively pentapeptides. At a pH of 7.0 the carboxyl groups of both acids are more likely to be deprotonated compared to pH 6.0 (Glu pK_aγCOOH_ = 4.3; Asp pK_aβCOOH_ = 3.90). Therefore, they provide a negative charge that is able to form ionic interactions with the cation of the ionic liquid Ammoeng 110™. Also, the partitioning behavior of free glycine is influenced by the pH value. At a pH of 6.0, glycine is in its tautomeric state and has negligible charge (Fig. 4). At a pH value of 7.0, glycine gets out of the tautomeric state and therefore the relative charge of the molecule gets up and the glycine concentration in the IL-rich upper phase of the ATPS gets increased. Combining all these findings and results it was concluded that the strong electrostatic/ionic interactions between the amino acids respectively the pentapeptides and the ionic liquid Ammoeng 110™ are the main driving forces for the extraction of the molecules into the IL-rich upper phase of the investigated IL-ATPS.

Furthermore, the usage of EZ:faast™-Kit from Phenomenex for the quantification of amino acids and peptides in the presence of the IL Ammoeng 110™ is in combination with the LC–MS measurement an easy and reliable method.

At last, the partitioning of amino acids and peptides in IL-based ATPS represents a complex process which most likely can only be explained by a combination of several driving forces (Oppermann et al. 2011). A broad array of factors, including peptide size, interactions of different salts with each other and the phase-forming compounds, and the interactions of the ionic liquid with amino acids and peptides have to be taken into account. These results are in line with the findings of Dreyer et al. (2009), who investigated the partitioning behavior of different proteins in IL based ATPS as well as with the conclusions of Heimer et al. (2014) who modeled the interaction between the IL [C_2_mim][OAc] and the peptide µ-SIIIA (Dreyer et al. 2009; Heimer et al. 2014).

Still, since partitioning is a surface-dependent phenomenon, investigations were focused on surface-related amino acid respectively peptide properties such as charge and hydrophobicity. Surface in this context means the surface of the phases in contact, but also the surface of the individual molecules. Especially in larger species such as peptides different surface properties will result due to peptide sequence and folding. Only a very low dependence between the amino acids or peptides hydrophobicity and the partition coefficient was found. Nevertheless, the presented results show that electrostatic respectively ionic interactions between the ionic liquid and the amino acids or peptides have a strong impact on their partitioning behavior. Therefore it was concluded, that these interactions are the main driving forces for amino acid and peptide extraction into the IL-rich upper phase of the system.

## Abbreviations

ATPS: aqueous two-phase system
IL: ionic liquid
LC–MS: liquid chromatography–mass spectrometry
pH: potential of hydrogen
SPE: solid-phase extraction
RP-HPLC: reverse-phase high performance liquid chromatography
SD: standard solution
V: volumes
h: height
d: diameter
HARG: homoarginine
Met-d3: methionine-d3
HPHE: homophenylalanine
Arg: arginine
Ser or S: serine
Gly or G: glycine
Phe or F: phenylalanine
Trp: tryptophan
Leu or L: leucine
Tyr: tyrosine
Glu or E: glutamic acid
His or H: histidine
Trp: tryptophane
D: aspartic acid
LTQ: linear trap quadrupole
m/z: mass-to-charge ratio
MS: mass spectrometry
D: distribution ratio
c: concentration
pI: isoelectric point
Ac: acetylated
[C_4_mim][Cl]: 1-n-butyl-3-methylimidazolium chloride
[N_4444_][Cl]: tetrabutylammonium chloride
[C_2_mim][OAc]: 1-ethyl-3-methyl-imidazolium acetate
Fmoc: fluorenylmethyloxycarbonyl
HBTU: (2-(1H-benzotriazol-1-yl)v-1,1,3,3-tetramethyluronium-hexafluorophosphat
AE110: Ammoeng 110™
pKa: logarithmic acid dissociation constant
Log D: distribution ratios
